# AMICA DEMENTIA SCREENING TOOLS ADAPTATION: AN INTERCULTURAL CONSENSUS APPROACH

**DOI:** 10.1093/geroni/igae098.0894

**Published:** 2024-12-31

**Authors:** Melissa Blind, Sheamus Cavanaugh, Nathania Tsosie, Carrie Trojanczyk, Nickolas Lambrou, Carey Gleason, Tassy Parker, Kristen Jacklin

**Affiliations:** University of Minnesota Medical School Duluth, Duluth, Minnesota, United States; University of Minnesota Medical School Duluth, Duluth, Minnesota, United States; University of New Mexico, Albuquerque, New Mexico, United States; University of Wisconsin Madison, Madison, Wisconsin, United States; University of Wisconsin Madison, Madison, Wisconsin, United States; University of Wisconsin Madison, Madison, Wisconsin, United States; University of New Mexico, Albuquerque, New Mexico, United States; University of Minnesota Medical School, Duluth, Minnesota, United States

## Abstract

Existing clinical tools for assessing dementia demonstrate varying degrees of cultural, educational and language biases, and are not validated for use with Indigenous populations in the US. The American Indigenous Cognitive Assessment (AMICA) project aims to develop and validate a culturally appropriate dementia evaluation toolkit that consists of four assessments. Participating Indigenous populations include the Red Lake Nation in Minnesota; an urban Indigenous population receiving services at the First Nations Community HealthSource in Albuquerque, New Mexico; and the Oneida Nation in Wisconsin. Our goal is to create one set of assessments that is agreed upon through intercultural consensus. Assessment adaptation across the three sites will focus on the Canadian Indigenous Cognitive Assessment (CICA), Kimberley Indigenous Cognitive Assessment (KICA) Carer, KICA Depression Scale, and KICA Activities of Daily Living (ADLs). We use a community-based participatory research (CBPR) approach and the Two-Eyed Seeing integrated knowledge framework to engage Tribal and community collaborators. In this paper, we detail the process of convening Indigenous Knowledge Advisory Groups (IKAGs) at each site and an Assessment Expert Panel (AEP), building a team science approach, and facilitating tool adaptation with communities. We provide an update on the CICA adaptation and offer lessons learned that inform culturally relevant and safe adaptation of the remaining tools. Finally, we report on our process for achieving intercultural consensus in developing a single set of dementia assessment tools for Indigenous peoples across the US.

